# The Transcription Factor Ets1 Influences Axonal Growth via Regulation of Lcn2

**DOI:** 10.1007/s12035-023-03616-0

**Published:** 2023-09-06

**Authors:** Miao Gu, Xiaodi Li, Ronghua Wu, Xiao Cheng, Songlin Zhou, Xiaosong Gu

**Affiliations:** 1 School of Medicine & Holistic Integrative Medicine, Nanjing University of Chinese Medicine, Nanjing, Jiangsu China; 2 School of Basic Medical Sciences, Hebei Key Laboratory of Nerve Injury and Repair, Chengde Medical University, Chengde, Hebei China; 3 Key Laboratory of Neuroregeneration of Jiangsu and Ministry of Education, Co-Innovation Center of Neuroregeneration, NMPA Key Laboratory for Research and Evaluation of Tissue Engineering Technology Products, Nantong University, Nantong, Jiangsu China

**Keywords:** Axonal growth, Axon regeneration, Ets1, Lcn2, Spinal cord development, Transcription factor

## Abstract

Transcription factors are essential for the development and regeneration of the nervous system. The current study investigated key regulatory transcription factors in rat spinal cord development via RNA sequencing. The hub gene Ets1 was highly expressed in the spinal cord during the embryonic period, and then its expression decreased during spinal cord development. Knockdown of Ets1 significantly increased the axonal growth of cultured spinal cord neurons. Luciferase reporter assays and chromatin immunoprecipitation assays indicated that Ets1 could directly bind to the Lcn2 promoter and positively regulate Lcn2 transcription. In conclusion, these findings provide the first direct evidence that Ets1 regulates axon growth by controlling Lcn2 expression, and Ets1 may be a novel therapeutic target for axon regeneration in the central nervous system.

## Introduction

The intrinsic regenerative capacity of neurons, which is lost in a development-dependent manner, is crucial for spinal cord regeneration [1]. It is strongest during the embryonic stage, decreases in infancy, and almost disappears in adulthood [2]. Therefore, one potential therapeutic strategy for spinal cord injury is to enhance the intrinsic regenerative capacity of spinal cord neurons [3–5].

Understanding the mechanisms that regulate spinal cord development may provide insight into spinal cord regeneration [6]. We have previously performed bulk spinal cord mRNA sequencing from the embryonic stage to adulthood to determine the temporal expression patterns of key genes in rat development [7]. Interestingly, we found that 100 transcription factors were highly expressed at embryonic day 11 during spinal cord development. Recent studies suggest that transcription factors are involved in axon regeneration, indicating that they may modulate neuronal functions.

The E-26 transformation-specific (Ets) family of transcription factors consists of 85-amino acid DNA-binding domains that are evolutionarily conserved throughout the metazoa [8]. They are primarily involved in crucial biological processes, including cell proliferation, cell migration, development, cell differentiation, angiogenesis, and cell cycle [9–12]. Ets1, a member of this family, plays a critical role in many normal physiological processes, such as promoting embryonic vasculogenesis and angiogenesis in zebrafish [13]. In the immune system, Ets1 suppresses the differentiation of type 2 T follicular helper cells, thereby halting the onset of systemic lupus erythematosus [14]. Ets1 is also a crucial regulator of human natural killer cell development and terminal differentiation [15]. However, whether Ets1 is expressed in the nervous system and the functions of Ets1 involved in the development and regeneration of the nervous system remain elusive and require further investigation. Our previous RNA sequencing data indicated that Ets1 mRNA is highly expressed in rat spinal cord [7].

The primary aim of the current study was to investigate the temporal expression and biological functions of Ets1 in detail. Changes in Ets1 expression in the rat spinal cord during development were evident, indicating that Ets1 plays a suppressive role in axon regeneration via interaction with Lcn2. This study highlights the crucial role of Ets1 in axon regeneration. Knockdown of Ets1 may be a novel molecular therapy for axon regeneration in the central nervous system.

## Material and Methods

### Animals

Sprague–Dawley (SD) rats at different developmental stages were used in this study. Embryonic day 11 (E11d), E13d, E14d, E18d (weight: 330–350 g), postnatal day 1 (P1d) (weight: 7 g), postnatal week 1 (P1w) (weight: 15 g), and P8w (weight: 200–230 g) were obtained from the Laboratory Animals Center of Nantong University, China. Animal procedures were approved by the Animal Ethics Committee of Nantong University, China (approval No. IACUC20201010-001). The rats were housed in a room with a temperature of 22 ± 2 °C, a humidity of 50–65%, and a 12:12-h light/dark cycle. They had free access to food and water.

### Bioinformatic Analysis

Previously archived RNA sequencing data of the spinal cord at different developmental stages (E11d, E14d, E18d, P1d, P1w, P8w) were downloaded from the NCBI database PRJNA505253 and screened for essential transcription factors involved in central nervous system development. The Ingenuity Pathway Analysis (IPA) online software (https://digitalinsights.qiagen.com/, Ingenuity Systems, USA) was used to construct a regulatory network of transcription factors.

### Immunofluorescence Staining

The spinal cords of rats at each time point were fixed in 4% paraformaldehyde at 4 °C overnight. Samples were cross-sectioned at a thickness of 12 µm using a cryostat microtome (Leica, Germany). Primary cultured neuronal cells were fixed with 4% paraformaldehyde for immunocytochemistry. Spinal cord sections and primary cultured neuronal cells were blocked with Immunol Staining Blocking Buffer (Beyotime, China) for 1 h at 37 °C. The spinal cord sections were incubated with primary rabbit polyclonal anti-Ets1 antibody (1:200, ab26096, Abcam, USA), mouse monoclonal anti-Nestin antibody (1:200; MAB353, Millipore, USA), and mouse monoclonal anti-NeuN antibody (1:200; MAB377, Millipore, USA) at 4 °C for 12 h. Primary cultured neuronal cells were incubated with primary mouse monoclonal anti-Tuj1 antibody (1:1000, ab7751, Abcam, USA) and rabbit polyclonal anti-Ets1 antibody. After being rinsed thrice with phosphate-buffered saline, the samples were incubated with the secondary antibodies Alexa Fluor 488 donkey anti-mouse IgG (1:500, SA00006-5, Proteintech, China) and Cy3 goat anti-rabbit IgG (1:500, SA00009-2, Proteintech, China) for 2 h at room temperature. The nuclei were stained with DAPI (ab104139, Abcam, USA). Each experiment was replicated three times. The images were visualized via confocal laser microscopy (Leica, Germany) and fluorescence microscopy (Stellaris5, Leica, Germany).

### Primary Neuronal Cell Culture and Transfection

The spinal cords were isolated from day 13 embryos of pregnant Sprague–Dawley rats as described previously (16,17). After enzymolysis with 0.125% trypsin–EDTA for 30 min at 37 °C, the digestion process was terminated with a complete medium containing DMEM/F12 (Corning, USA), 10% fetal bovine serum (Gibco, USA), 1% penicillin–streptomycin (Beyotime, China), and 1% glutamine (Beyotime, China). Subsequently, neuronal cells were transfected with Ets1 siRNA (target sequence GCAGAAAGAGGATGTGAAA, Ribobio, China), Lcn2 siRNA (target sequence CUGGGCCUCAAGGAUAACATT, Ribobi, China), or control siRNA (target sequence UUCUCCGAACGUGUCACGUTT, Ribobio, China) at a final concentration of 200 nM using a NEPA21 electrical transfection instrument at 275 V for 0.7 ms following the manufacturer’s instructions, to reduce the expression of Ets1 and Lcn2. After transfection, the neurons were cultured in the neuronal culture medium, consisting of Neurobasal medium (Gibco, USA), 2% B27 supplement (Gibco, USA), 1% penicillin–streptomycin, and 1% glutamine at 37 °C in a humidified incubator with 5% CO_2_ for 48 h for immunohistochemistry.

### Quantitative Real-Time PCR

Total RNA was isolated from the spinal cords and neuronal cells using an RNA-Quick Purification Kit (Yishan Biotechnology Co., China), then reverse transcribed using the PrimeScript RT reagent Kit with gDNA Eraser (TaKaRa, China). Quantitative real-time PCR (qRT-PCR) was conducted using FastStart Essential DNA Green Master (Roche, USA) on a StepOne real-time PCR system (Applied Biosystems, USA). Specific primers were synthesized by Genscript Biotech (Nanjing, China) and are listed in Table 1. Gene quantification was performed using the comparative 2^−ΔΔCt^ method with glyceraldehyde-3-phosphate dehydrogenase (Gapdh) as the internal control.

**Table 1:** The sequences of primers used in this study

qRT-PCR primers	Forward (5′–3′)	Reverse (5′–3′)
Ets1	CTGACTTGCTTCTCCCCAGG	TGCCGCTACATTTCCAGTGT
Lcn2	GGGCTGTCCGATGAACTGAA	TGCTTGGTGGAATCATGGCT
Apob	GTTACGGCTGGAACCACTGA	ACATCAACGGAGGAAGCCAG
Psd4	GGAAGACAACCTACAGCCCC	GTTCCACATCAGAGTCGCCA
Nags	GGGACCTGCAAACGTTGTTC	ATTTACAGGGCCGGTGATGG
GAPDH	AACGACCCCTTCATTGAC	TCCACGACATACTCAGCAC

### Microfluid Chamber

Neurons were seeded onto the somatic side of a poly-l-lysine-coated microfluidic chamber (SND 150, Xona Microfluidics, Temecula, USA) at a density of 5 × 10^4^ cells per cm^2^ and incubated for 4 h. After cell attachment, 200 nM Ets1 siRNA or control siRNA was added to the somatic side of the microfluidic chamber. After 4 days of culture, axons entering the axonal side were injured using 0.08 MPa vacuum suction (GL-802A, Kylin-Bell, China) three times for 30 s each time. Injured axons were allowed to grow for 24 h, then Tuj1 immunofluorescence staining was performed and images were acquired via a fluorescence microscope.

### RNA Sequencing

Transcriptome sequencing of neuronal cells transfected with control siRNA or Ets1 siRNA was performed using Illumina HiSeq2500 by Novogene Biotechnology Co. (Beijing, China). Gene expression levels were determined using the fragments per kilobase of transcript per million mapped reads (FPKM) method. Fold changes of > 2 or <  − 2 were deemed to indicate differentially expressed genes, and a false discovery rate of < 0.05 compared to corresponding controls was set. Differentially expressed genes were categorized via Gene Ontology (GO) and Kyoto Encyclopedia of Genes and Genomes (KEGG) analysis using OmicShare bioinformatic tools (https://www.omicshare.com/tools/, China). Sequencing data were deposited in the database with the accession number PRJNA938264.

### Prediction of Binding Site

To identify potential binding sites of Ets1 transcription factors, the promoter region approximately 2000 bp upstream of the transcription start site of the Lcn2 gene was predicted using the JASPAR online database (https://jaspar.genereg.net/).

### Luciferase Reporter Assay

The coding sequence of Ets1 was inserted into the GV141 vector, and the promoter sequence 2000 bp upstream of the Lcn2 transcription start site was inserted into the GV238 vector (GeneChem, Shanghai, China). After transfection of HEK-293 T cells, luciferase reporter assay detection was conducted 48 h later using the Dual-Luciferase Reporter Assay System (Promega) on a BioTek Synergy 2. Renilla luciferase activity was used as an internal control.

### Chromatin Immunoprecipitation Assay

Chromatin immunoprecipitation (ChIP) assays were performed using a SimpleChIP® Plus Enzymatic Chromatin IP Kit (Cell Signaling Technology) according to the manufacturer’s instructions. Briefly, B35 cells were lysed, and chromatin immunoprecipitation was performed using an anti-Ets1 polyclonal antibody (14069, Cell Signaling Technology). Eluted DNA fragments were analyzed via PCR and quantitative PCR. The primers for the Lcn2 promoter (5′–3′) were as follows: GAGCTACAAGGGGCTGGAA (forward) and TCCCTGGATGATGAAAGAACA (reverse).

### Statistical Analysis

Numerical data are presented as means ± standard error of the mean. Student’s *t*-test was used for comparisons between two groups, and comparisons between multiple groups were performed using one-way analysis of variance (ANOVA) followed by Dunnett’s multiple comparisons test. *p* < 0.05 was considered statistically significant.

## Results

### Ets1 Is Highly Expressed During Spinal Cord Development in Rats

**Fig. 1:**
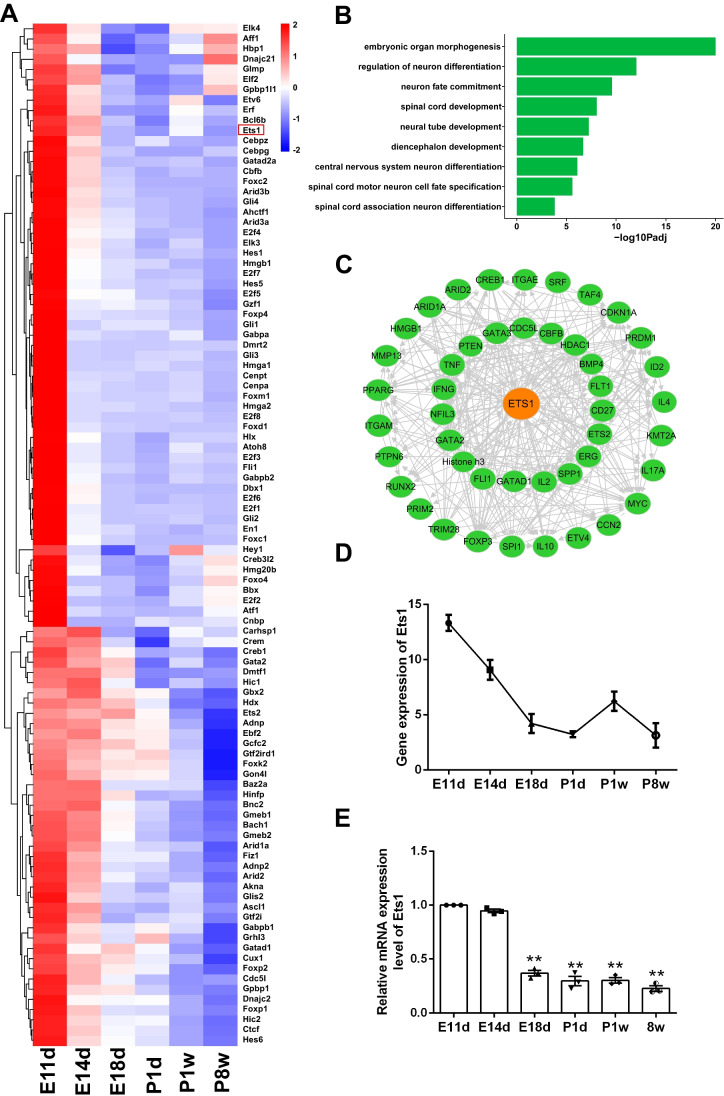
Expression profile of Ets1 in rat spinal cord development. **A** Hierarchical clustering of transcription factor expression profiles in rat spinal cord development at different time points. **B** Top terms yielded by Gene Ontology analysis of transcription factors. **C** Ingenuity Pathway Analysis of Ets1 as a hub gene in rat spinal cord development. **D** Fragments per kilobase of exon model per million mapped reads (FPKM) Ets1 expression trends derived from RNA sequencing during rat spinal cord development at different time points. **E** Relative Ets1 mRNA expression during rat spinal cord development at different time points normalized to E11d. *n* = 3 independent experiments. **p < 0.01 vs. E11d control

**Fig. 2:**
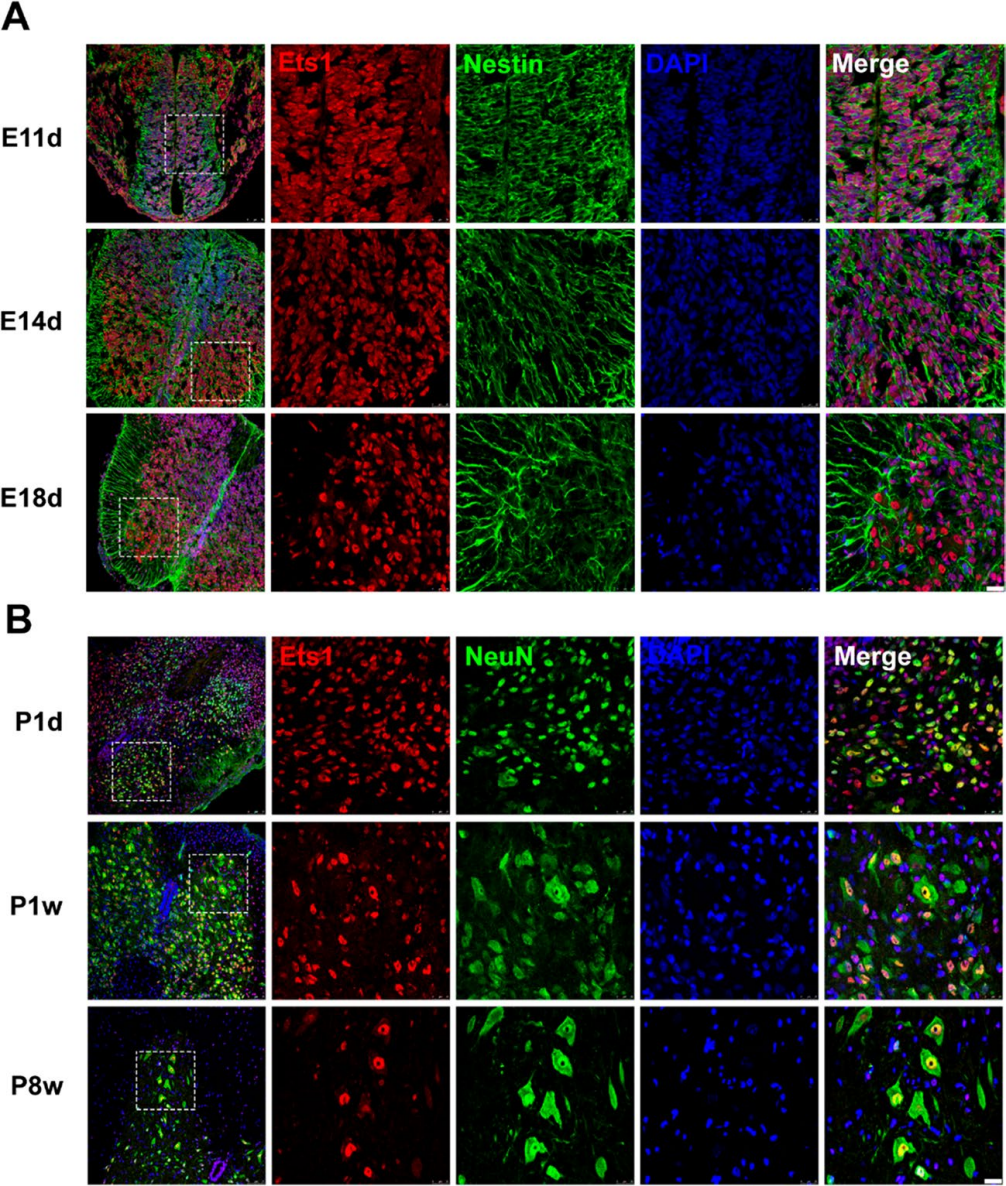
Expression of Ets1 in rat spinal cord. **A** Ets1 and Nestin double staining in rat spinal cord at E11d, E14d, and E18d. Red indicates Ets1, green indicates Nestin, and blue indicates DAPI. Boxed areas within the panels on the left are displayed at higher magnification in the boxes on the right. Scale bars = 25 µm. **B** Ets1 and NeuN double staining in rat spinal cord at P1d, P1w, and P8w. Red indicates Ets1, green indicates NeuN, and blue indicates DAPI. Scale bars = 25 µm

A total of 100 transcription factors showed high expression in the spinal cord at E11d (Fig. 1A). GO analysis revealed enrichment of transcription factors involved in neuron fate commitment, spinal cord motor neuronal cell fate specification, and spinal cord-associated neuron differentiation (Fig. 1B). These findings suggest that these transcription factors may play a crucial role in spinal cord development. For further investigation, we selected the hub gene Ets1, which was predicted by Ingenuity Pathway Analysis (Fig. 1C). RNA sequencing analysis confirmed that Ets1 had high expression levels at E11d, which decreased during spinal cord development (Fig. 1D). qRT-PCR also indicated a similar expression pattern (Fig. 1E). Immunofluorescence results demonstrated that Ets1 was primarily observed in spinal cord neural stem cells identified by Nestin during the embryonic period and in neurons identified by NeuN after birth (Fig. 2).

### Ets1 Inhibits Axonal Growth of Spinal Cord Neurons

**Fig. 3:**
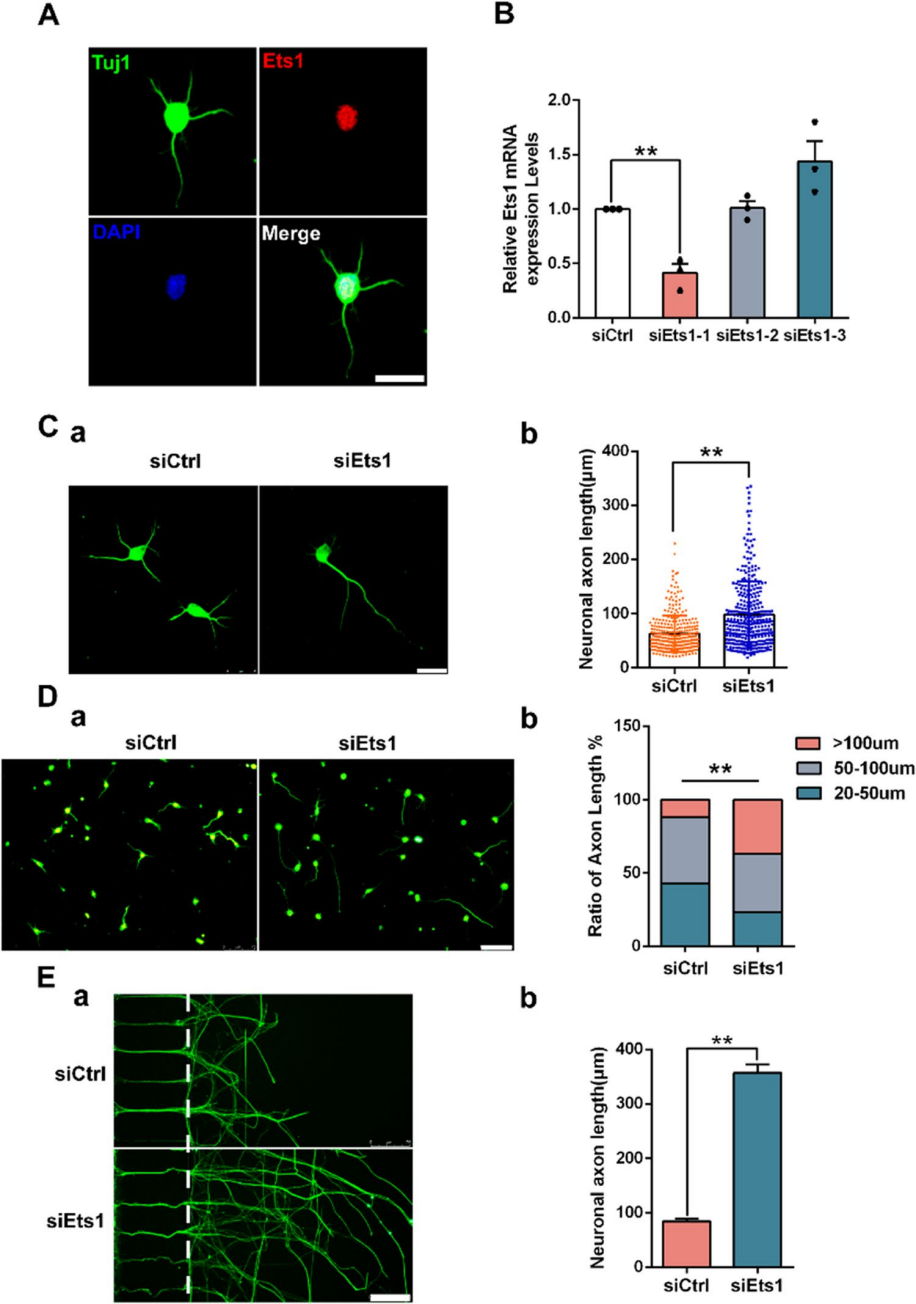
Localization of Ets1 in cultured spinal cord neurons, and promotion of axonal growth by Ets1 knockdown. **A** Immunofluorescence staining showing that Ets1 (red) co-localizes with spinal cord neurons (green) in the nucleus. Tuj1 indicates spinal cord neurons and DAPI indicates the nucleus. Scale bar = 25 µm. **B** Ets1 mRNA expression in spinal cord neurons after transfection with control siRNA or Ets1 siRNA. siCtrl represents control siRNA and siEts1-1, siEts1-2, and siEts1-3 represent three Ets1 siRNAs. *n* = 3 independent experiments. **p < 0.01. **C** Effect of Ets1 siRNA treatment on axonal growth. (a) Representative images of Ets1 knockdown in spinal cord neurons stained with Tuj1 antibody. Scale bar = 50 µm. (b) The longest axon lengths per spinal cord neuron after Ets1 siRNA were quantified. *n* = 3 independent experiments. **p < 0.01 vs. control siRNA. **D** (a) Representative images of Tuj1 immunostaining in neurons treated with control or Ets1 siRNA for 48 h. Scale bar = 75 µm. (b) Ratios of axon lengths per spinal cord neuron after Ets1 siRNA. Axon length 20–50 µm: siCtrl = 43%, siEts1 = 23%; axon length of 50–100 µm: siCtrl = 45%, siEts1 = 40%; axon length > 100 µm: siCtrl = 12%, siEts1 = 37%. *n* = 300 neurons. **p < 0.01. **E** (a) Tuj1 staining of regenerated axons in microfluidic chambers. Green indicates Tuj1. Scale bar = 75 µm. (b) Lengths of regenerated axons in neurons treated with control siRNA or Ets1 siRNA. *n* = 3 independent experiments. **p < 0.01

Immunofluorescence indicated that Ets1 was predominantly localized in the nucleus of neurons (Fig. 3A). In qRT-PCR analysis of cultured spinal cord neurons transfected with three Ets1 siRNAs or control siRNA, Ets1 expression was significantly decreased in the Ets1 siRNA group compared to the control group (Fig. 3B). Neurite outgrowth assays showed that transfection with Ets1 siRNA markedly promoted axonal growth of neurons (Fig. 3C). The mean length of axons in the Ets1 siRNA group was 56% greater than that in the control group (*n* = 300, *p* < 0.01). Axonal growth phenotype after Ets1 siRNA treatment was analyzed. Neurons were divided into three categories based on axonal length [16, 17]: 20–50 µm, 50–100 µm, and > 100 µm. Compared to the control group, neurons in the Ets1 siRNA group exhibited higher ratios in the > 100 µm and 50–100 µm categories (Fig. 3D). In experiments with injured axons, the regenerated lengths of injured axons increased approximately threefold after 24 h of culture and treatment with Ets1 siRNA (Fig. 3E). Taken together, these results indicate that Ets1 suppresses axonal growth of spinal cord neurons.

### Ets1 Modulates Neuronal Cell Metabolism

**Fig. 4:**
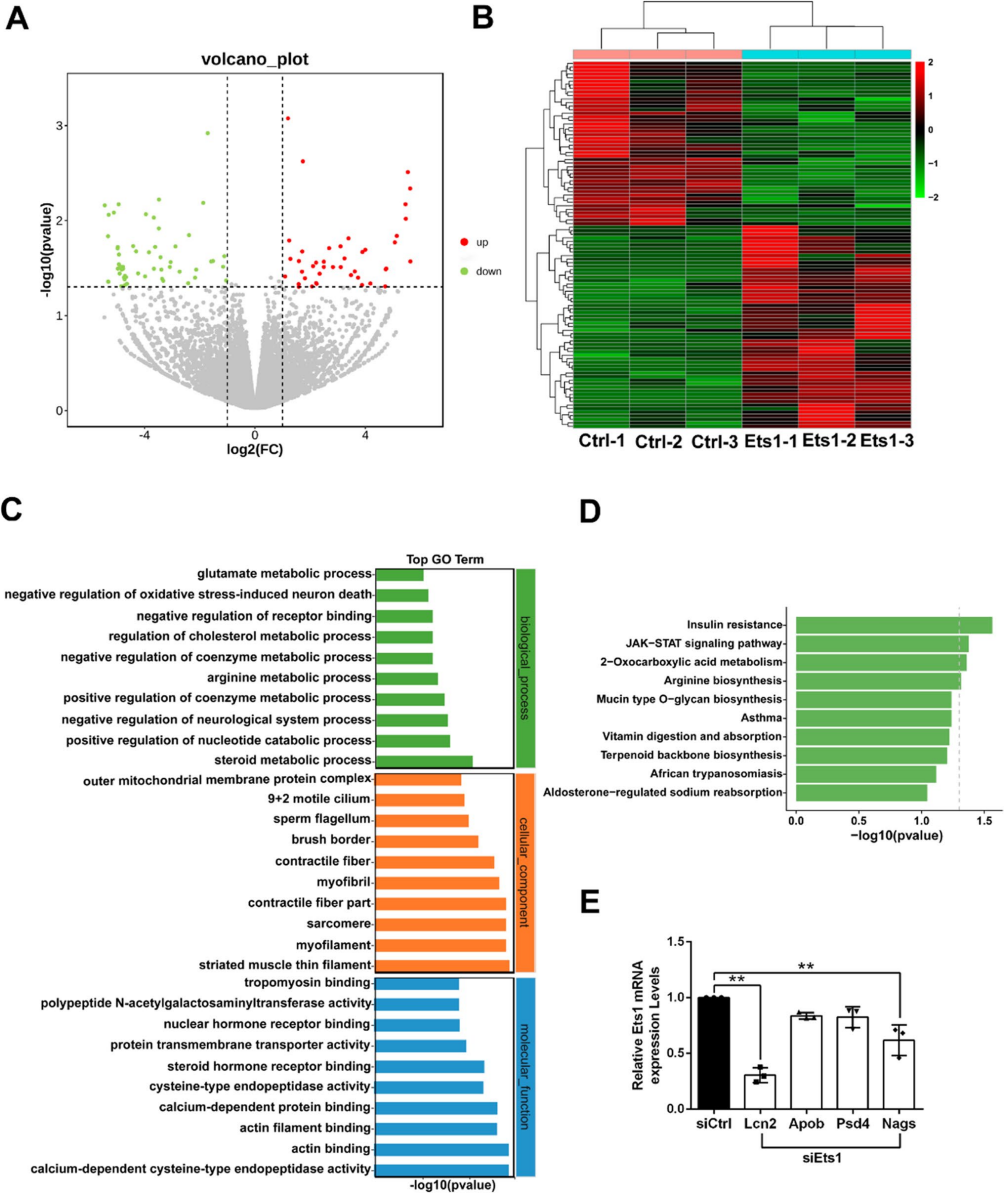
Ets1 influences the biological activities of spinal cord neurons. **A** Scatter plots of differentially expressed genes in spinal cord neurons transfected with Ets1 siRNA. Red represents significantly upregulated genes, and green represents significantly downregulated genes. **B** Heatmap of the expression levels of differentially expressed genes. Red indicates high expression levels; green indicates low expression levels. **C** GO biological process, cellular component, and molecular function terms of differentially expressed genes. **D** KEGG pathways of differentially expressed genes. **E** RNA sequencing qRT-PCR results of four genes. *n* = 3 independent experiments. **p < 0.01 vs. control siRNA

To decode the molecular changes induced by Ets1 knockdown, neuronal cells transfected with control siRNA or Ets1 siRNA were subjected to RNA sequencing. A total of 103 differentially expressed genes were identified between the control group and the Ets1 siRNA group, with 46 genes upregulated and 57 genes downregulated (Fig. 4A). The expression levels of differentially expressed genes were shown in the heatmap (Fig. 4B). GO analysis indicated that differentially expressed genes were enriched in various processes, including regulation of steroid metabolic processing (GO:0019218), positive regulation of coenzyme metabolic processes (GO:0051197), arginine metabolic processing (GO:0006525), negative regulation of coenzyme metabolic processing (GO:0051198), glutamate metabolic processing (GO:0006536), and regulation of cholesterol metabolic processing (GO:0090181) (Fig. 4C). The KEGG pathway analysis indicated that differentially expressed genes were enriched in insulin resistance, the JAK-STAT signaling pathway, 2-oxocarboxylic acid metabolism, and arginine biosynthesis (Fig. 4D). We selected high fold change genes (Lcn2, Apob, Psd4, and Nags) from the 103 differentially expressed genes. In qRT-PCR experiments, the mRNA levels of Lcn2, Apob, Psd4, and Nags were significantly decreased after Ets1 knockdown, compared to the control group. These results were consistent with the expression trends determined by RNA sequencing (Fig. 4E).

### Ets1 Targets Lcn2 and Inhibits Axonal Growth of Spinal Cord Neurons

**Fig. 5:**
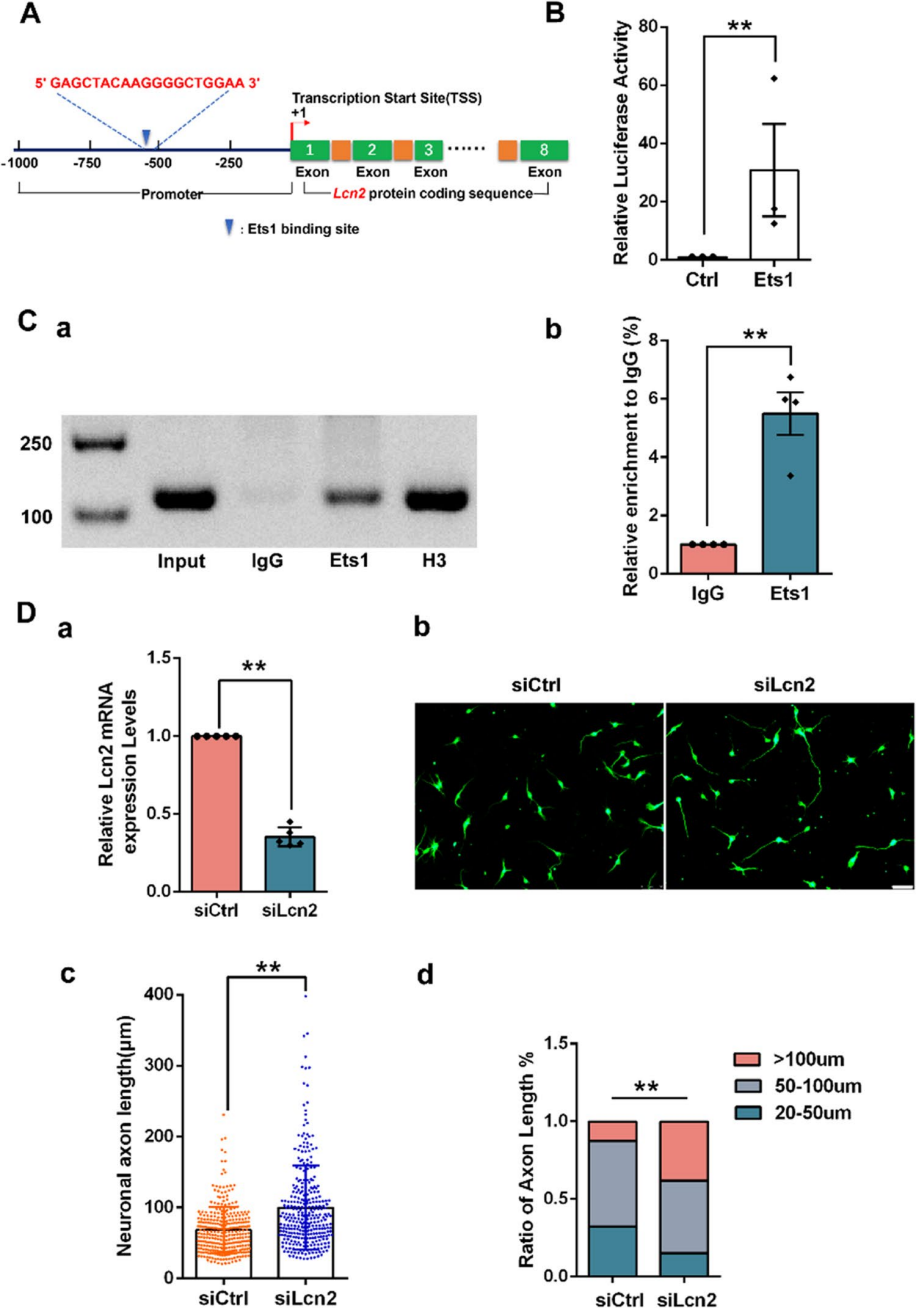
Ets1 targets Lcn2 and affects spinal cord neuron axonal growth. **A** Prediction of a potential downstream Ets1 target gene. **B** Luciferase activity driven by Lcn2 promoter in HEK-293 T cells after Ets1 overexpression (luciferase reporter assay). **p < 0.01 vs. control. **C** Chromatin immunoprecipitation assays showed that Ets1 occupied the promoter of endogenous Lcn2. The enrichment of binding sites was detected by ChIP qPCR. **p < 0.01 vs. control. **D** (a) Lcn2 mRNA expression in spinal cord neurons after transfection with control siRNA or Lcn2 siRNA. *n* = 3 independent experiments. **p < 0.01 vs. control siRNA. (b) Representative images of Tuj1 immunostaining in neurons treated with control siRNA or Lcn2 siRNA for 48 h. Scale bar = 75 µm. (c) The longest axon lengths per spinal cord neuron after Lcn2 siRNA. *n* = 3 independent experiments. **p < 0.01 vs. control siRNA. (d) Ratios of axon lengths per spinal cord neuron after Lcn2 siRNA. Axon length 20–49 µm: siCtrl = 32%, siEts1 = 15%; axon length 50–100 µm: siCtrl = 55%, siEts1 = 47%; axon length > 100 µm: siCtrl = 13%, siEts1 = 38%. *n* = 300 neurons. **p < 0.01

JASPAR analysis was performed using RNA sequencing data, predicting the presence of Ets1 binding sites in the promoters of the Lcn2 gene (Fig. 5A). qRT-PCR analysis indicated that Lcn2 mRNA levels were decreased dramatically after Ets1 knockdown in neuronal cells (Fig. 4E). Luciferase assays revealed that the luciferase activity of the Lcn2 reporter was significantly upregulated by Ets1 overexpression, indicating that the Ets1 binding sequences serve as positive regulatory elements for Lcn2 transcription (Fig. 5B). ChIP assays confirmed the direct association of Ets1 with Lcn2 promoters (Fig. 5C). To investigate whether reduced Lcn2 expression due to Ets1 knockdown influenced axon growth in neuronal cells, Lcn2 siRNA was transfected into neuronal cells. qRT-PCR results showed reduced Lcn2 mRNA expression in neuronal cells transfected with Lcn2 siRNA (Fig. 5D-a). Knockdown of Lcn2 promoted the growth of neuron axons (Fig. 5D-b, 5D-c), with a significant increase observed in the proportion of axons longer than 100 µm (Fig. 5D-d). Overall, these findings indicate that the downregulation of Ets1 in neuronal cells negatively affects Lcn2 expression, thereby promoting axonal growth in neurons.

## Discussion

Transcription factors play a crucial role in spinal cord injury. Recent studies have suggested that transcription factors, such as ATF3 [18, 19], Sox11 [20], STAT3 [21], and Smad1 [22–24], are involved in axon regeneration, indicating their potential importance in neuronal functions. The current study aimed to investigate the effects of the transcription factor Ets1, which shows differential expression during spinal cord development, on the axonal growth of neuronal cells.

Ets1 has been associated with various conditions, including hepatocellular carcinoma [25, 26], healthy aging [27], congenital heart defects [28], and arthritis [29]. Our bioinformatic analyses of transcription factors during spinal cord development identified Ets1 as a critical upstream regulatory gene, indicating its potential influence on neuronal cells. In the current study, we analyzed the expression profiles of Ets1 in the spinal cord during rat development and found that Ets1 expression was significantly downregulated during spinal cord development. To investigate the role of Ets1, we comprehensively investigated the influence of Ets1 on spinal cord neurons by transfecting primary cultured neuronal cells with Ets1 siRNA. We identified an inhibitory effect of Ets1 on axonal growth in spinal cord neurons. Future studies may explore the application of Ets1 antagomir as a potential treatment for central nervous system injury.

Mechanistic studies revealed that Ets1 knockdown reduced the expression levels of Lcn2, suggesting that Lcn2 is a downstream target of Ets1. Lcn2 is involved in multiple cellular processes, including cellular uptake of iron, apoptosis, suppression of invasiveness and metastasis, and glial activation. It also plays a crucial role in brain injury and recovery after ischemic and hemorrhagic stroke [30, 31]. Another study has shown that Lcn2 may exert damaging effects after cerebral ischemia by inducing classical activation of astrocytes [32]. Reactive astrocytes are reported to use NOX signaling to stimulate Lcn2 expression and secretion, and blocking astrocytic NHE1 activity promotes the reduction of Lcn2-mediated neurotoxicity after stroke [33]. Recent studies have investigated the effects of Lcn2 on Alzheimer’s disease [34, 35], spinal cord injury [36], perioperative neurocognitive disorders [37], and diabetic retinopathy [38]. In the current study, luciferase assays and ChIP assays revealed that Ets1 bound to the Lcn2 promoter and regulated its activity. Knockdown of Lcn2 promoted axonal growth in spinal cord neurons and had the same effects as Ets1 knockdown on axonal growth in neuronal cells. These findings of the present study suggest that Ets1 regulates axonal growth in spinal cord neurons through the target gene Lcn2. In summary, the present study demonstrates that Ets1 may modulate axonal growth of spinal cord neurons through interaction with Lcn2.

Interestingly, although Ets1 expression is low in the adult spinal cord, the axonal growth capacity is also low. The expression of genes in adulthood does not correspond to axonal growth capacity. For example, deletion of Krüppel-like factor-4 (Klf4) has been reported to promote axonal regeneration in retinal ganglion cells, despite low Klf4 expression in adulthood [39]. This is similar to the situation with Ets1 in the current study. On the other hand, inhibition of Gas5 expression promoted axonal growth in dorsal root ganglion (DRG) neurons, and the Gas5 expression was high in adult dorsal root ganglia [40]. The role of Ets1 was only demonstrated in vitro in the present study, and its role in vivo still needs to be confirmed. Furthermore, the ability of Ets1 to suppress axon elongation could be further investigated to gain a more comprehensive understanding of its functional roles.

Overall, these findings highlight the inhibitory role of Ets1 in the axon growth of neuronal cells, identify Lcn2 as a downstream functional target of Ets1, and expand our understanding of the molecular changes occurring during spinal cord development and regeneration. This study clarifies the key role of Ets1 in spinal cord neurons and provides compelling evidence for the potential use of Ets1-based molecular therapy as a novel method for treating nerve injury in the central nervous system.

## Data Availability

The datasets used and analyzed during the current study are available from the corresponding author upon reasonable request.
